# Does AI's touch diminish the artistry of scientific writing or elevate it?

**DOI:** 10.1186/s13054-023-04634-z

**Published:** 2023-09-11

**Authors:** Mugdha Gandhi, Madhura Gandhi

**Affiliations:** 1 Independent Researcher, Pune, India; 2 Central Research Facility, Dr. D. Y. Patil Medical College, Hospital and Research Centre, Dr. D. Y. Patil Vidyapeeth, Pune, Maharashtra India

Dear Editor,

We are writing to express our insights on the increasing use of artificial intelligence (AI) and tools in scientific writing and its potential impact on the quality of research publications in recent years. As an enthusiastic observer of technological advancements and a researcher, we believe it is essential to shed light on how AI has reshaped the way we conduct and disseminate research and its impact on the integrity of scholarly work.

Traditionally, scientific writing has been considered an art where scientists spend significant time and effort, which involves meticulous research, thoughtful analysis and language skills to convey their findings accurately.

AI, particularly machine learning and natural language processing algorithms, can assist in generating abstracts, drafts, proofreading, suggesting citations, optimizing language to improve the readability of scientific manuscripts and even the peer-review process [1]. It has also demonstrated efficiency in handling extensive datasets, identifying patterns and leading to more robust and evidence-based findings. This not only accelerates the pace of research but also reduces the burden on researchers and reviewers to some extent [2].

Additionally, AI-powered recommendation tools can analyze a broad spectrum of researchers’ existing publications and citations to suggest diverse areas of research based on expertise and foster collaboration among scholars in the world, thereby enabling scientists to broaden their knowledge base and make informed decisions.

Despite these remarkable benefits, our concern lies in the certain challenges associated with the ethical use of AI in research publications. The widespread adoption of AI tools may affect the art of scientific writing and creativity in conveying research findings, limiting researchers’ critical thinking and analytical skills and leading to a reduction in the diversity of research approaches, which adds richness to the scientific literature [3]. While these tools raise concerns about potential misuse or plagiarism, when used ethically, they can help researchers save time and effort, thereby increasing productivity and enhancing the overall quality of research output. Moreover, data privacy and potential biases present in AI algorithms must be addressed rigorously to ensure the responsible and equitable use of AI technologies.

The overuse of AI could lead to a decrease in demand for skilled professionals in various fields, resulting in a lack of transparency in the decision-making process and a loss of human touch. There is a need for continuous human oversight and critical analysis to prevent the misleading information generated by AI systems and to avoid the deprivation of essential qualitative insights that human expertise can provide for tasks such as peer review and data analysis [4].

In conclusion, the impact of AI on research publications has been transformative, revolutionizing the way we conduct and publish scientific knowledge. As we move forward, the scientific community needs to find a balance between leveraging AI tools for efficiency and preserving the indispensable art of scientific writing to maintain the integrity and credibility of scholarly publications and nurture collaborative research efforts for the betterment of humanity.

## Data Availability

Not applicable.
